# Supplementary material for: DTLR-CS: Deep tensor low rank channel cross fusion neural network for reproductive cell segmentation
Source: PLoS One. 2023 Nov 30;18(11):e0294727. doi: 10.1371/journal.pone.0294727 (PMC10688749; doi:10.1371/journal.pone.0294727)
Supplement: Attachment — Submitted filename: Response to Reviewer.doc [file pone.0294727.s001.doc]

Response to Reviewer’s Comments on

DTLR-CS: Deep Tensor Low Rank Channel Cross Fusion Neural Network for Reproductive Cell Segmentation

Thanks to the reviewers for their valuable comments, which were very helpful to our paper. We have studied reviewer’s comments carefully and have tried our best to revise our manuscript according to the comments, in particular, the datasets and figures have been improved, relevant working sections have been added, and the language has been embellished.

The main changes are as follows:

1) We have reorganized the presentation of the datasets, explaining the dataset source and data restriction.

2) We corrected mistakes in grammar, spelling, formulas and figures in the article, and added some required references.

3) We have reworked the conclusion section to make it more succinct and easier to read.

All the revised parts except references are colored blue in the revised manuscript.

Replies to Reviewer 1’ s comments

1. Kindly explain regarding the data restriction this project.

Answer: Thank you very much for the comment. We have made revisions to the subsection “Datasets”, providing a detailed explanation of the data limitations in this paper.

In order to ensure the credibility and effectiveness of the study, we have introduced certain data restrictions during the process of data collection. Firstly, the dataset exclusively comprises 2018 germ cell images, maintaining a consistent timeframe to reduce potential variations due to changes in practices, equipment, or patient demographics. Secondly, the dataset encompasses diverse germ cells without restricting the cause of infertility or patient age, capturing a wide range of real-world scenarios to enhance result generalizability and representativeness. Furthermore, a significant data limitation involves the minimum ovum count per patient, specifically cases with eight or more ovum acquired. This stipulation ensures comprehensive germ cell development views per data point, reducing outlier impact and enhancing statistical analysis robustness. These restrictions establish a well-defined scope for the study, facilitating a focused and comprehensive analysis of germ cell behavior and development within the context of infertility and IVF procedures.

2. Kindly transform the bullet points into paragraph format and consider to write the sub heading according to standard scientific paper sub heading.

Answer: Thanks for your comments. We have reformatted the bullet points into a paragraph format and ensure that the subheadings are written according to the standard subheading style commonly found in scientific papers.

Replies to Reviewer 2’ s comments

1. The Source of the dataset taken should be clearly mentioned in detail in the subsection “Datasets”.

Answer: Thanks for pointing out the issue. As follows, we have restructured the content of the "Datasets" section and added information about the sources of the datasets.

The dataset used in this paper is a self-constructed dataset, utilizing reproductive cell images from the Reproductive Medicine Center of Zhongda Hospital affiliated with Southeast University in 2018 to train and evaluate our method.

2. The authors have tried to identify the gaps or flaws in the related works done in the “Introduction” section. It is suggested to cite more references for the statements furnished there.

Answer: Thanks for your comments. We have added the following reference in the “Introduction”section:

[1]Valiuškaitė, Viktorija, et al. "Deep learning based evaluation of spermatozoid motility for artificial insemination." Sensors 21.1 (2020): 72.

[2]Vicar, Tomas, et al. "Cell segmentation methods for label-free contrast microscopy: review and comprehensive comparison." BMC bioinformatics 20.1 (2019): 1-25.

[3]Khairandish, Mohammad Omid, et al. "A hybrid CNN-SVM threshold segmentation approach for tumor detection and classification of MRI brain images." Irbm 43.4 (2022): 290-299.

[4]Lee, Jungbeom, et al. "Ficklenet: Weakly and semi-supervised semantic image segmentation using stochastic inference." Proceedings of the IEEE/CVF Conference on Computer Vision and Pattern Recognition. 2019.

[5]Guo, Qinpeng, et al. "A method of blasted rock image segmentation based on improved watershed algorithm." Scientific Reports 12.1 (2022): 7143.

[6] Chen, Xu, et al. "Learning active contour models for medical image segmentation." Proceedings of the IEEE/CVF conference on computer vision and pattern recognition. 2019.

[7]Minaee, Shervin, et al. "Image segmentation using deep learning: A survey." IEEE transactions on pattern analysis and machine intelligence 44.7 (2021): 3523-3542.

[8]Kayalibay, Baris, Grady Jensen, and Patrick van der Smagt. "CNN-based segmentation of medical imaging data." arXiv preprint arXiv:1701.03056 (2017).

[9]Ren, Yupeng, et al. "Image-based concrete crack detection in tunnels using deep fully convolutional networks." Construction and Building Materials 234 (2020): 117367.

3. The Figure 4 and Figure 8 should be reconstructed to increase its visibility.

Answer: Thanks for pointing out the issue. As follows, we have modified Figure 4 and Figure 8 to make them clearer and increase their visibility.

4. It is suggested to include a subsection for Implementation and processing time of the proposed method.

Answer: Thanks for your comments. In the experimental part, we have added a subsection for Implementation and processing time. The content is as follows.

We use a 48 GB RAM NVIDIA A40 GPU card to implement our model in PyTorch. To prevent over-fitting, we use flipping for data enhancement. We train all our models for 40 epochs on 1 GPU with a batch size of 4, and our initial learning rate is set to 0.001. We use the Adam optimiser to optimise our network. The Adam optimiser is simple to implement and computationally efficient. In addition, the update of the parameters using the Adam optimiser is not affected by the scaling transformation of the gradient, while the learning rate can be automatically adjusted, making it suitable for scenarios with large scale data and parameters. Training the model with the Deep Tensor Low Rank Channel Cross Fusion Neural Network took the shortest run time with the lowest trainable number of parameters improvement in the model performance.

5. Please rewrite the “Conclusion” section to make it succinct.

Answer: Thanks for your comments. As follows, we have reworked the “Conclusion” section to make it more succinct in terms of clarity of expression.

In this paper, we propose a segmentation model based on U-Net that can efficiently achieve contextual information and reduce semantic differences, which obtains the contextual information contained in the high-rank tensor by low-ranking the tensor. Then the model passes the spatial information of the shallow layers to the decoder by fusing features from between different layers of the encoder. Finally, it connects the encoder and decoder by a cross-fusion connection module in order to recover the full spatial resolution. Experimental results show that our method is more competitive than existing segmentation methods.
